# Pharmaceutics: A New Open-Access Journal for All Those Interested in Designing Medicines

**DOI:** 10.3390/pharmaceutics1010001

**Published:** 2009-10-02

**Authors:** Yvonne Perrie, Afzal R. Mohammed

**Affiliations:** Aston Pharmacy School, School of Life and Health Sciences, Aston University, Birmingham. B4 7ET, UK

We know the many hurdles that face us when we look to deliver a drug, starting from the basic characteristics of the drug (its solubility, stability, absorption and biodistribution), to overcoming the physiological barriers faced in reaching the target site, and to maintaining the concentration within the therapeutic window. In addition we must also remember the patient needs in this – is it a child that needs a liquid dosage form? Is it someone having to take multiple doses in a day? Do we need a rapid onset of action in a convenient format? Will people find it convenient to take the drug in the format we are presenting to them – or are there alternative options? 

Henry Ford is rightly recognised for his development of mass production which brought affordable cars; his comment that ‘customers could have a car painted any colour that he wants so long as it is black’, certainly showed how we could streamline manufacturing and improve efficiency. However we all like, and often need, more refinement in the things we use. Personalised medicines is currently a popular topic, but actually what we need is individualised healthcare where we tailor all aspects of the healthcare provision to the needs of an individual; starting from the therapeutic drug action, through to the formulation platform. So whilst there is still a strong and continued requirement for new drugs, much of these problems can be overcome with effective formulation. 

*Pharmaceutics* aims to help to widen discussions and open new research considerations in terms of dosage forms and their design; it is an open access journal which provides an advanced forum for the science and technology of pharmaceutics and biopharmaceutics. What is particularly exciting about this new journal is that we want to encourage researchers to publish their work in as much detail as possible, so that people can easily reproduce methods in their own laboratories and authors can discuss in greater detail possible mechanisms behind results so to open up the research to wider discussion. There are, in addition, unique features of this journal including the option for computed data or files regarding the full details of the experimental procedure, if unable to be published in a normal way, to be deposited as supplementary material – a key feature in supporting data sharing within our community.

To help us achieve our aim, we have an excellent Editorial Board to advise us of all the exciting current and future trends in the area of pharmaceutics and biopharmaceutics and we will soon be high-lighting this range of expertise in a forthcoming special issue – ‘What’s on Board’, where we get to read in detail the research that is being undertaken in the laboratories of our Editorial Board members. However we very much look forward to hearing all about the research going on in the many pharmaceutics laboratories across the world, and publishing your latest findings.

